# Supplementary material for: Parallel algorithms for phylogenetic inference under a structured coalescent approximation
Source: bioRxiv. 2025 Sep 24:2025.09.22.677844. Preprint. [Version 1] doi: 10.1101/2025.09.22.677844 (PMC12485945; doi:10.1101/2025.09.22.677844)
Supplement: Supplement 1 [file NIHPP2025.09.22.677844v1-supplement-1.pdf]

bioRxiv preprint doi: https://doi.org/10.1101/2025.09.22.677844; this version posted September 24, 2025. The copyright holder for this preprint (which was not certified by peer review) is the author/funder, who has granted bioRxiv a license to display the preprint in perpetuity. It is made available under aCC-BY 4.0 International license.

Supplementary Materials

Additional benchmark results

To isolate the performance gains from our algorithmic restructuring versus parallelization benefits, we performed a single-threaded comparison of structured coalescent calculations between BEAST X CPU and the BASTA package in BEAST 2.7.7. Supplementary Figure S1 demonstrates that even without any parallelization, BEAST X CPU achieves substantial speedup factors of 8.2× for EBLV (51 taxa, 3 demes), 7.0× for ZIKV (283 taxa, 22 demes), and 7.0× for PEDV (756 taxa, 26 demes). These performance improvements arise solely from our fundamental restructuring of the SCA likelihood computation, which eliminates redundant calculations through efficient caching of partial likelihoods, optimizes memory access patterns, and streamlines the peeling algorithm. The consistent 7-8× speedup across datasets of varying complexity indicates that our algorithmic optimizations provide robust baseline improvements independent of hardware parallelization. This single-threaded performance gain is particularly important as it benefits all users regardless of their computational resources, while the additional parallelization capabilities shown in the main text (Figure 1) can provide further acceleration on multi-core systems.

bioRxiv preprint doi: https://doi.org/10.1101/2025.09.22.677844; this version posted September 24, 2025. The copyright holder for this preprint (which was not certified by peer review) is the author/funder, who has granted bioRxiv a license to display the preprint in perpetuity. It is made available under aCC-BY 4.0 International license.

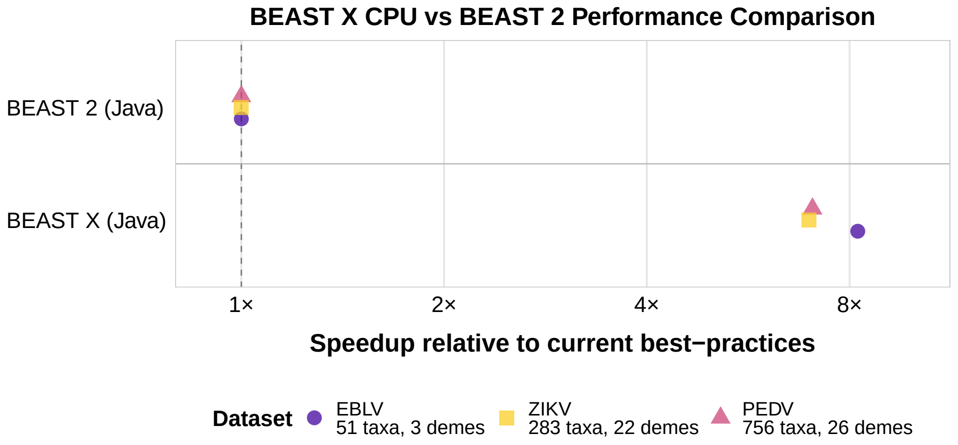

Supplementary Figure S1: Performance comparison between single-threaded BEAST X CPU and BEAST 2.7.7 for structured coalescent approximation (SCA) analyses. The plot shows speedup factors on a log scale for three viral datasets: EBLV (51 taxa, 3 geographic states), ZIKV (283 taxa, 22 states), and PEDV (756 taxa, 26 states). BEAST X CPU achieves 7.0–8.2× speedup over BASTA package in BEAST 2.7.7 through algorithmic restructuring alone, without any parallelization. The vertical dashed line at 1× represents baseline BEAST 2.7.7 performance.
